# Stopping azithromycin mass drug administration for trachoma: A systematic review

**DOI:** 10.1371/journal.pntd.0009491

**Published:** 2021-07-08

**Authors:** Hamidah Mahmud, Emma Landskroner, Abdou Amza, Solomon Aragie, William W. Godwin, Anna de Hostos Barth, Kieran S. O’Brien, Thomas M. Lietman, Catherine E. Oldenburg

**Affiliations:** 1 University of California, San Francisco School of Medicine, San Francisco, California, United States of America; 2 Francis I. Proctor Foundation, University of California, San Francisco, California, United States of America; 3 Programme National de Santé Oculaire, Niamey, Niger; 4 The Carter Center Ethiopia, Addis Ababa, Ethiopia; 5 Department of Ophthalmology, University of California, San Francisco, California, United States of America; 6 Department of Epidemiology & Biostatistics, University of California, San Francisco, California, United States of America; RTI International, UNITED REPUBLIC OF TANZANIA

## Abstract

The World Health Organization (WHO) recommends continuing azithromycin mass drug administration (MDA) for trachoma until endemic regions drop below 5% prevalence of active trachoma in children aged 1–9 years. Azithromycin targets the ocular strains of *Chlamydia trachomatis* that cause trachoma. Regions with low prevalence of active trachoma may have little if any ocular chlamydia, and, thus, may not benefit from azithromycin treatment. Understanding what happens to active trachoma and ocular chlamydia prevalence after stopping azithromycin MDA may improve future treatment decisions. We systematically reviewed published evidence for community prevalence of both active trachoma and ocular chlamydia after cessation of azithromycin distribution. We searched electronic databases for all peer-reviewed studies published before May 2020 that included at least 2 post-MDA surveillance surveys of ocular chlamydia and/or the active trachoma marker, trachomatous inflammation–follicular (TF) prevalence. We assessed trends in the prevalence of both indicators over time after stopping azithromycin MDA. Of 140 identified studies, 21 met inclusion criteria and were used for qualitative synthesis. Post-MDA, we found a gradual increase in ocular chlamydia infection prevalence over time, while TF prevalence generally gradually declined. Ocular chlamydia infection may be a better measurement tool compared to TF for detecting trachoma recrudescence in communities after stopping azithromycin MDA. These findings may guide future trachoma treatment and surveillance efforts.

## Author summary

Trachoma, caused by repeated infections with ocular *Chlamydia trachomatis*, substantially contributes to the global burden of blindness. Community-wide distribution of the oral antibiotic azithromycin in trachoma endemic regions has contributed to significant decline in the prevalence of both ocular chlamydia infection and clinical findings of active trachoma. After azithromycin mass drug administration (MDA) stops, both ocular chlamydia and active trachoma can return. Our systematic review finds that ocular chlamydia infection may return to communities faster than signs of active trachoma, which may help better understand the utility of different trachoma indicators for post-MDA surveillance.

## Introduction

Trachoma causes an estimated 3% of the world’s blindness, with 84 million active cases as of 2019 [1]. Repeat ocular *Chlamydia trachomatis* infections cause tarsoconjunctival scarring, which retracts the upper eyelid inward. The resulting eyelash abrasion leads to corneal opacity, and, ultimately, blindness [2–4]. In many regions previously endemic for trachoma, prevalence has drastically declined, due, at least in part, to mass drug administration (MDA) with azithromycin [5,6]. The World Health Organization (WHO) Alliance for the Global Elimination of Trachoma by 2020 (GET 2020) recommends continuing annual oral mass azithromycin distribution until affected regions drop below 5% prevalence of active trachoma (trachomatous inflammation–follicular, TF) in children aged 1 to 9 years [1]. These guidelines were developed based on expert consensus rather than empirical data, and understanding whether recrudescence of infection or active trachoma occurs after stopping azithromycin MDA could guide future treatment programming.

Azithromycin specifically targets ocular chlamydia infection and has been shown to rapidly reduce ocular chlamydia prevalence in treated communities. One 2009 study in Ethiopia had a baseline mean prevalence of ocular chlamydia infection of 48.9% prior to the start of MDA, which decreased to 5.4% 2 months after stopping MDA. However, the mean prevalence increased to 7.9% by 6 months after cessation of MDA [7]. The correlation between TF and infection is poor after repeated rounds of treatment, and TF is a lagging indicator for infection as inflammation takes more time to clear that the infection itself [8–10]. Measurement of ocular chlamydia may be a better indicator for detecting recrudescence of infection than active trachoma [11–14,15,16].

Although WHO uses a 5% TF threshold for decision-making related to stopping azithromycin MDA, districts with TF prevalence above the threshold may have very low or zero prevalence of ocular chlamydia [17]. If true, then additional rounds of azithromycin distribution may not lead to substantial declines in either TF or ocular chlamydia, and stopping azithromycin MDA should not lead to appreciable recrudescence. Furthermore, azithromycin MDA may be stopped due to political insecurity or natural disasters at any TF threshold [18]. Understanding trends in both TF and ocular chlamydia prevalence at any point in which azithromycin MDA is stopped may give valuable evidence for programs experiencing treatment interruptions. In this systematic review, we therefore sought to review all published studies reporting ocular chlamydia and/or TF prevalence at a pretreatment time point and at least 2 posttreatment time points and to evaluate any recrudescence of TF or chlamydia infection after stopping azithromycin MDA. We included studies of any length, with any baseline TF and ocular chlamydia prevalence, conducted at any time, in any country on community-wide distribution of oral azithromycin for the prevention and treatment of trachoma available in several of the larger scientific literature databases.

## Methods

### Search strategy and selection criteria

We systematically reviewed all published literature available in English without date restrictions. We searched the Cochrane Library, Embase, Medline, and Web of Science databases for studies published from database inception until May 15, 2019. We also searched all conference abstracts available online from the American Society for Tropical Medicine and Hygiene (ASTMH). The search was updated on May 18, 2020 to capture recently published literature.

We included the terms “trachoma” and “azithromycin” in all electronic searches. We used variations of the search string (“Trachoma” [Mesh] OR trachoma) AND (“Azithromycin” [Mesh] OR azithromycin OR Zithromax) when appropriate. From all retrieved citations, we removed duplicates, screened titles and abstracts for relevance, and reviewed full articles that met our inclusion criteria. Two independent researchers (HM and EL) screened titles and abstracts and reviewed full articles, and a third (CEO) adjudicated discrepancies.

We included all primary studies on community-wide distribution of oral azithromycin for trachoma that measured prevalence of ocular chlamydia and/or active trachoma before and after azithromycin MDA. Eligible studies included 1 pretreatment time point and at least 2 distinct posttreatment time points. We included studies that concurrently used topical tetracycline for trachoma treatment if azithromycin MDA was also noted. We excluded studies on the use of azithromycin MDA for purposes other than the prevention and treatment of trachoma, those without pretreatment and with less than 2 posttreatment time points, those without sufficient information on the number of individuals or villages tested, those evaluating individual-level (rather than community-level) azithromycin treatment, and studies of mathematical models, surveillance reports, and review articles.

### Outcomes and data extraction and quality

HM extracted data from all full articles included. Data were extracted directly into the REDCap electronic data capture tools hosted at the University of California, San Francisco [19,20]. The main outcomes of interest were the prevalence of active trachoma, using each paper’s definition as TF or TF/TI, and ocular chlamydia infection before and after community level azithromycin MDA. Other variables extracted from each study included study design, geographic location, sample sizes, and duration and frequency of azithromycin MDA. We used the Cochrane Collaboration’s tool for assessing risk of bias in randomized trials and the ROBINS-I tool for assessing risk of bias in nonrandomized trials to assess quality [21,22]. We assessed risk of bias for trachoma prevalence from original publications by rating all included studies as having high, moderate, low, or unclear risk of bias in each of the domains specified by the ROBINS-I tool [22]. Because our qualitative synthesis was focused on understanding time trends rather than the effect of a specific exposure, we considered risk of bias due to confounding to be low for all studies. Measurement error due to heterogeneity in graders exists for all studies due to the nature of field grading. We considered measurement error to be low if the study masked laboratory personnel to time points and processed samples for ocular chlamydia in a random order. Studies that only included TF measurements were considered to be at moderate risk of measurement error.

### Data synthesis and statistical analysis

Due to heterogeneity across study designs and time points for measuring trachoma outcomes, we did not undertake a formal meta-analysis. We conducted a qualitative synthesis of all studies, summarizing ocular trachoma and TF prevalence over time in each included study. To assess recrudescence in individual studies, we plotted the ocular chlamydia and TF prevalence at the first and second post-MDA time points. We also plotted the mean TF or ocular chlamydia prevalence in each study for all available post-MDA discontinuation time points to assess trends over time in trachoma prevalence after stopping MDA. We completed and reported this systematic review according to Preferred Reporting Items for Systematic reviews and Meta-Analyses (PRISMA) 2009 guidelines and have included the checklist in the Supporting information. The study protocol was registered in PROSPERO (CRD42021140510) and protocols.io (dx.doi.org/10.17504/protocols.io.bsaenabe). The protocol is also included in the Supporting information.

## Results

**Fig 1:**
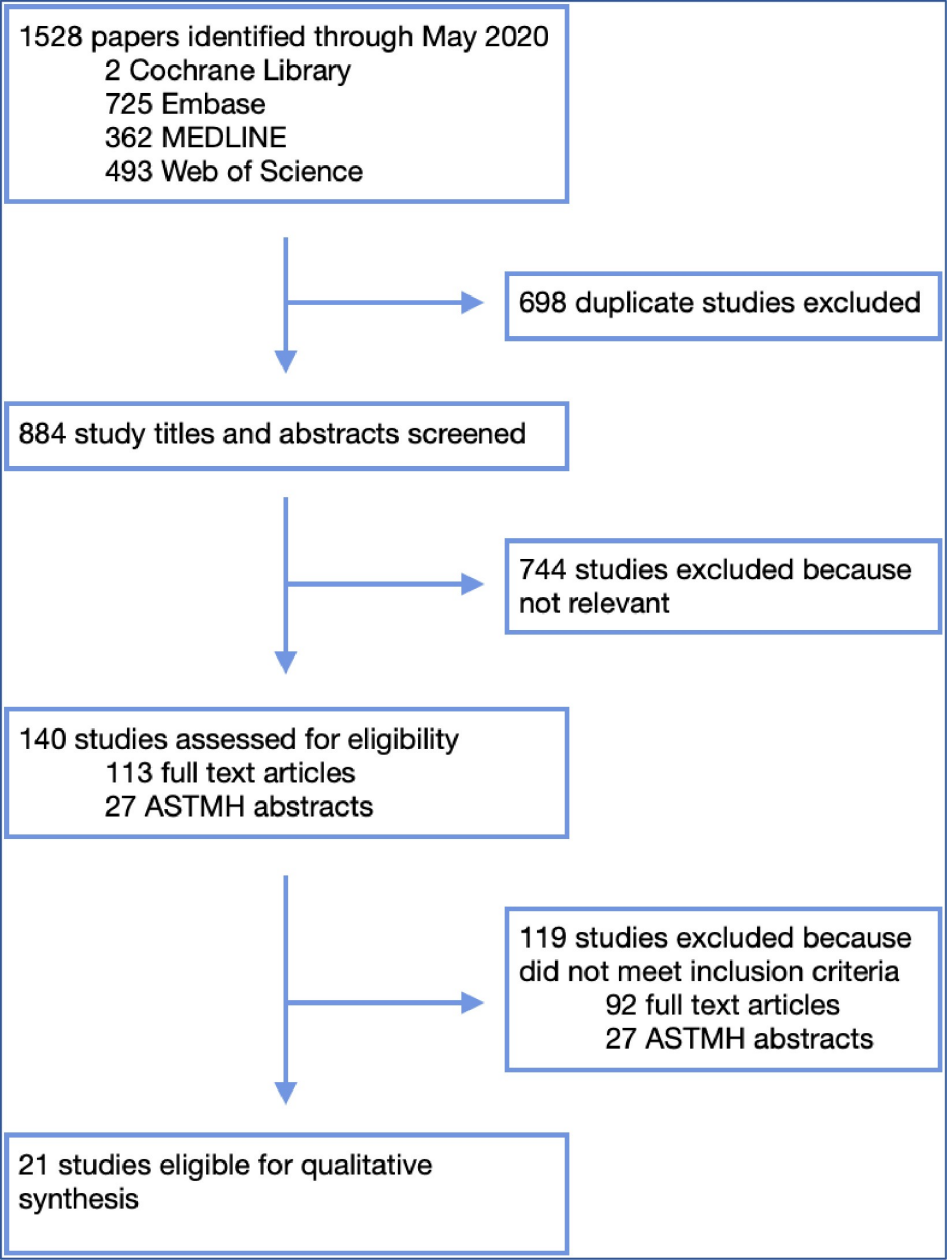
Flow diagram of the study selection process. ASTMH, American Society for Tropical Medicine and Hygiene.

We identified 1,528 papers through our database search (Fig 1). After duplicate removal, we screened the title and abstract of 884 studies. A total of 140 studies met eligibility for full review including 113 full text articles and 27 ASTMH abstracts. Moreover, 119 studies were then excluded for not meeting inclusion criteria, often for insufficient post-MDA time points or not following a community-wide treatment model. Our qualitative synthesis covered data from a total of 21 published, full-text articles.

We analyzed 21 papers published between 1999 and 2019, including 15 cohort studies and 6 randomized trials (Table 1). A total of 9 studies reported from Tanzania, 6 from Ethiopia, 4 from the Gambia, and 1 each from Australia, Egypt, Mali, and Nepal. Moreover, 452 communities in total from 7 countries were included in this analysis. Furthermore, 13 studies had a single distribution of MDA, while others varied in treatment frequency from weekly to annual models. Treatment duration varied from a single MDA time point to 4 years. A total of 4 studies reported only ocular chlamydia prevalence, 4 reported only active TF levels, and 13 reported both ocular chlamydia and active TF. Pretreatment prevalence of ocular chlamydia infection ranged from 0% to 70.7% and of active trachoma (TF or TF/TI) ranged from 4.9% to 91.6%. The time between the 2 posttreatment time points ranged from 2 to 42 months with an average of 6 months.

**Table 1:** Characteristics of included studies.

Study	Author	Country	Year	Design	Communities treated	Treatment frequency	Treatment duration	Endpoint measured	Pre-discontinuation active TF prevalence	Pre-discontinuation ocular chlamydia infection prevalence	Time between first 2 post-MDA d/c surveys
1	Broman et al. [23]	Tanzania	2006	Cohort study	1	Single		Both	76.6%	68.3%	2 months
2	Burton et al. [24]	The Gambia	2010	Cohort study	14	Single		Both	15.4%	9.7%	2 months
3	Burton et al. [25]	The Gambia	2005	Cohort study	14	Single		Both	8%	7%	2 months
4	Chidambaram et al. [6]	Ethiopia	2006	Cohort study	8	Single		Ocular chlamydia		43.5%	2 months
5	Fraser-Hurt et al. [26]	The Gambia	2001	RCT	8	Weekly	3 weeks	TF	14.4%		2 months
6	Jha et al. [27]	Nepal	2002	Cohort study	18	Single		TF	19%		6 months
7	Keenan et al. [28]	Ethiopia	2010	Cohort study	24	Biannual	2 years		91.6%	63.5%	6 months
								Both			
					24	Biannual	3 years		74.9%	31.6%	6 months
8	Keenan et al. [15]	Ethiopia	2018	RCT	48	Annual	4 years			41.9%	12 months
								Ocular chlamydia			
					48	Biannual	4 years			38.3%	12 months
9	Lakew et al. [7]	Ethiopia	2009	RCT	40	Single		Ocular chlamydia		48.9%	2 months
10	Lakew et al. [29]	Ethiopia	2009	Cohort study	16	Biannual	2 years	Both	91.6%	63.5%	6 months
11	Lansingh et al. [30]	Australia	2010	Cohort study	2	Single		TF	49%		3 months
12	Melese et al. [31]	Ethiopia	2004	Cohort study	24			Ocular chlamydia		56.3%	2 months
13	Ramadhani et al. [32]	Tanzania	2019	Cohort study	3	Single		Both	34%	15%	3 months
14	Schachter et al. [13]	Egypt		RCT	1	Quarterly	1 year		50.2%	43.7%	4.5 months
		The Gambia	1999		1			Both	30.2%	37.2%	3 months
		Tanzania			1				43.3%	19.7%	3 months
15	Schémann et al. [33]	Mali	2007	Cohort study	7	Single		TF	23.7%		1 month
16	Solomon et al. [34]	Tanzania	2008	Cohort study	1	Annual	2 years	Both	9.5%	2.2%	18 months
17	Solomon et al. [5]	Tanzania	2004	Cohort study	1	Single		Both	20.4%	9.5%	2 months
18	West et al. [35]	Tanzania	2017	RCT	52	Single		Both	4.9%	3%	6 months
19	West et al. [36]	Tanzania	2007	Cohort study	1	Weekly	2 weeks	Both	53%	70.7%	42 months
20	West et al. [37]	Tanzania	2005	Cohort study	1	Single		Both	38%	57%	2 months
21	Wilson et al. [10]	Tanzania	2018	RCT	96	Single		Both	4.3%	0%	12 months

RCT, randomized controlled trial; TF, trachomatous inflammation–follicular.

**Fig 2:**
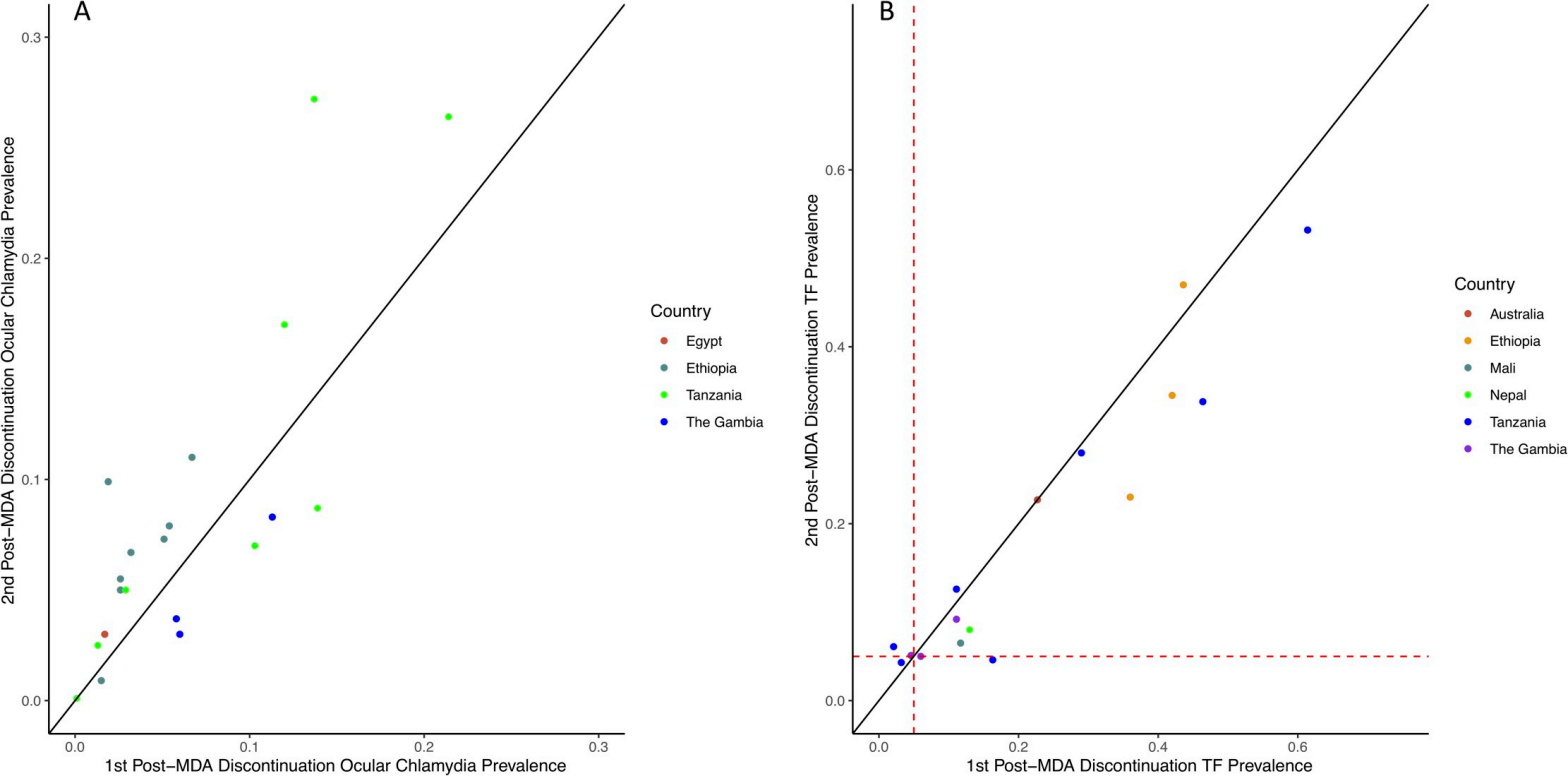
Prevalence of ocular chlamydia and active trachoma at first and second post-MDA discontinuation time points. Prevalence of ocular chlamydia (Fig 2A) and TF (Fig 2B) at first and second post-mass distribution administration of azithromycin surveys. The solid black line indicates 45° line. The red dashed line on panel (Fig 2B) indicates the TF control threshold (5%). MDA, mass drug administration; TF, trachomatous inflammation–follicular.

**Fig 3:**
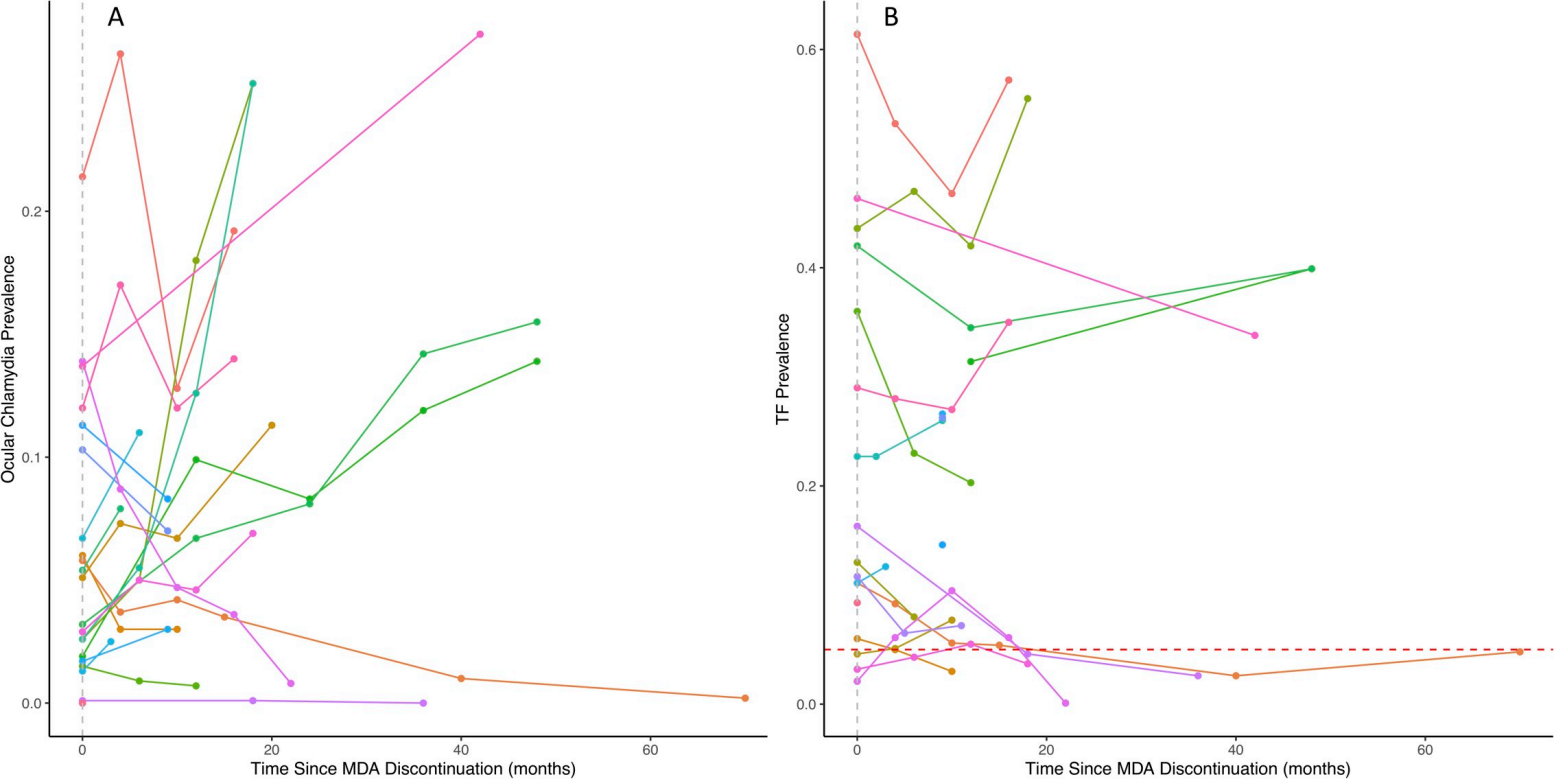
Prevalence of ocular chlamydia and active trachoma post-MDA discontinuation over time. Prevalence of ocular chlamydia (Fig 3A) and TF (Fig 3B) at each post-mass distribution administration of azithromycin time point (in months). The vertical gray dotted line indicates the first post-MDA time point for each included study. The red dashed line for Fig 3B indicates the 5% TF threshold. MDA, mass drug administration; TF, trachomatous inflammation–follicular.

The prevalence of ocular chlamydia increased from the first to second post-MDA surveillance survey per site in 13/20 (65%) studies (Fig 2A). Most studies conducted in Ethiopia and in those with high ocular chlamydia prevalence during the first post-MDA survey saw an increase in prevalence during the second post-MDA survey. Longitudinally, ocular chlamydia infection increased over time in most studies with the exception of those with low infection prevalence during the first post-MDA survey (Fig 3A).

TF prevalence remained relatively stable between the first and second post-MDA time points (Fig 2B), with TF prevalence declining between the surveys in most studies. TF prevalence tended to decline longitudinally in studies where the prevalence was less than 20% at the first post-MDA survey and tended to increase in studies with higher post-MDA TF prevalence (Fig 3B).

Although most studies were found to have overall low risk of bias, we considered risk of measurement error to be moderate in most studies as they did not explicitly state whether laboratory personnel were masked to time point (Table 2) or only includes measures of TF. Overall risk of bias was low for all included studies.

**Table 2:** Risk of bias assessment of included studies.

	Confounding	Selection bias	Classification of interventions	Deviation from intervention	Missing data	Measurement error	Reporting bias	Overall bias
Study 1	Low	Low	Low	Low	Low	Moderate	Low	Low
Study 2	Low	Low	Low	Low	Low	Moderate	Low	Low
Study 3	Low	Low	Low	Low	Low	Moderate	Low	Low
Study 4	Low	Low	Low	Low	Low	Moderate	Low	Low
Study 5	Low	Low	Low	Low	Low	Moderate	Low	Low
Study 6	Low	Low	Low	Low	Low	Moderate	Low	Low
Study 7	Low	Low	Low	Low	Low	Low	Low	Low
Study 8	Low	Low	Low	Low	Low	Moderate	Low	Low
Study 9	Low	Low	Low	Low	Low	Moderate	Low	Low
Study 10	Low	Low	Low	Low	Low	Low	Low	Low
Study 11	Low	Low	Low	Low	Low	Moderate	Low	Low
Study 12	Low	Low	Low	Low	Low	Low	Low	Low
Study 13	Low	Low	Low	Low	Low	Moderate	Low	Low
Study 14	Low	Low	Low	Low	Low	Low	Low	Low
Study 15	Low	Low	Low	Low	Low	Moderate	Low	Low
Study 16	Low	Low	Low	Low	Low	Moderate	Low	Low
Study 17	Low	Low	Low	Low	Low	Moderate	Low	Low
Study 18	Low	Low	Low	Low	Low	Low	Low	Low
Study 19	Low	Low	Low	Low	Low	Moderate	Low	Low
Study 20	Low	Low	Low	Low	Low	Moderate	Low	Low
Study 21	Low	Low	Low	Low	Low	Moderate	Low	Low

## Discussion

This systematic review synthesized data from 21 publications on trachoma prevalence after stopping azithromycin MDA. We found that studies of ocular chlamydia infection showed increases in post-MDA prevalence more often than those of active trachoma, even in studies with <10% prevalence of ocular chlamydia during the first post-MDA survey. Increases after stopping azithromycin MDA were seen less often with TF, with increases in the first post-MDA period in 5 studies. Even at higher baseline TF prevalence before MDA, TF prevalence did not always increase post-MDA. TF is a lagging indicator relative to ocular chlamydia; thus, it is possible that the time between the first 2 post-MDA surveys was not sufficient to show an increase in TF prevalence [21,22,38–40]. Longitudinally, communities with low TF prevalence during the first post-MDA survey tended to stay low, and those with higher prevalence were more likely to increase over time, suggesting a possible slow return in TF prevalence over time. More generally, there appears to be a slower reduction in TF prevalence during azithromycin mass drug distributions [7,14]. Communities with higher prevalence during the first post-MDA period likely have greater risk of resurgence than low-prevalence communities.

Many studies saw an increase in ocular chlamydia prevalence between the first and second post-MDA surveillance surveys (65%). Studies in Ethiopia and those with high first post-MDA survey prevalence had relatively higher ocular chlamydia prevalence during the second and third post-MDA time points. They also trended toward increased ocular chlamydia prevalence quicker than those studies with lower first post-MDA survey ocular chlamydia prevalence. This may be due to overall high prevalence of trachoma in some areas of Ethiopia and subsequent return of infection due to transmission from neighboring communities.

Based on our findings comparing TF and ocular chlamydia as markers for community prevalence of trachoma, ocular chlamydia may be a better indicator than signs of active trachoma for detecting recrudescence of infection. Although measurement of ocular chlamydia is more logistically challenging than TF, it may provide more sensitive information for detecting recrudescence following stopping azithromycin MDA. Trachoma programs could consider increasing use of ocular chlamydia measurement as a monitoring tool for trachoma recrudescence after stopping azithromycin MDA.

Most studies were conducted in areas with TF prevalence above the threshold for stopping MDA, both at baseline and after the first post-MDA discontinuation time point, which may limit the generalizability of these results to lower prevalence areas. Currently, WHO guidelines indicate annual azithromycin MDA until communities reach <5% TF prevalence [1]. The majority of studies included in this analysis had substantially higher TF prevalence at the time of stopping azithromycin MDA, and, thus, may be more likely to experience resurgence than those with lower baseline prevalence. TF more often increased in communities with post-MDA prevalence above 20%, suggesting that azithromycin MDA in areas with TF >20% should be continued in accordance with WHO guidelines. However, these findings demonstrate the potential repercussions of halting or delaying MDA in endemic areas due to funding issues, political unrest, and pandemics. Recent modeling studies have suggested that interruptions in MDA due to the Coronavirus Disease 2019 (COVID-19) pandemic may delay reaching elimination targets unless a catchup approach that includes increased frequency of azithromycin distribution is employed [18,41]. The results of this systematic review provide some empirical evidence that discontinuation of azithromycin MDA at higher trachoma prevalence levels will likely lead to increased prevalence of ocular chlamydia infection. However, waiting until TF is <5% to stop azithromycin MDA may not be necessary to achieve elimination in all settings, and earlier stopping of MDA would be an antibiotic-sparing approach that could minimize selection for antimicrobial resistance. Additional randomized controlled trial evidence evaluating when azithromycin distributions can be stopped would be helpful to provide guidance for ongoing azithromycin distribution [17,39,40]. Other possible approaches for evidence generation for trachoma control programs could include more frequent surveillance around the 5% threshold to detect recrudescence earlier, operational research to include a longer timeline for final surveillance (e.g., 3 to 5 years after stopping MDA) to understand longer-term trends post-MDA, and increasing utilization of ocular chlamydia measurement or other alternative indicators such as serology for monitoring trachoma recrudescence [42–44].

The results of this study must be considered in the context of several limitations. The heterogeneity of study design, geographical location, population samples, and time points limited our ability to conduct a formal meta-analysis. As previously mentioned, studies were predominantly conducted in higher prevalence settings. For example, our systematic review did not identify published research from countries recently validating elimination, including Morocco, Oman, and Ghana. Studies may be less likely to be conducted in lower prevalence areas, which could introduce some publication bias and affect generalizability of these results. Conclusions based on this systematic review may not be applicable to countries that have reached elimination. Some changes in prevalence that we observed post-MDA are fairly small. Individual studies included data from multiple communities, and we anticipate that some of this variability may be due to sampling variability across time points. However, our review was not able to statistically test for changes over time as we used single summary points, and, thus, these smaller changes may not be statistically significant and thus programmatically meaningful. Inherent risk of bias in community prevalence studies is a limitation of this systematic review. Our review of quality of studies showed that all included studies had low overall risk of bias, including low risk of confounding, selection, and reporting bias. However, risk of measurement error was considered to be moderate in majority of studies.

## Conclusions

Trachoma prevalence in trachoma endemic areas increases after stopping azithromycin MDA in some settings. We observed high heterogeneity in ocular chlamydia prevalence, but it may detect increases in trachoma following stopping MDA more quickly than TF.

**Key learning points**

- Mass drug administration (MDA) with the antibiotic azithromycin is recommended by the World Health Organization (WHO) for evaluation units (roughly equivalent to a district) until they reach 5% prevalence of active trachoma.
- Understanding what happens to the prevalence of active trachoma and the causative organism of trachoma, ocular *Chlamydia trachomatis*, when mass azithromycin distributions are stopped may help inform programs that are dealing with treatment interruptions or during surveillance for districts that have reached elimination.
- Trachoma prevalence increases when azithromycin distribution is stopped, especially in communities with higher pre-stopping prevalence.
- Ocular *C*. *trachomatis* may return more quickly that active trachoma.

**Top five papers**

1. Solomon AW, Holland MJ, Alexander NDE, Massae PA, Aguirre A, Natividad-Sancho A, et al. Mass treatment with single-dose azithromycin for trachoma. N Engl J Med. 2004;351(19):1962–71.
2. Schachter J, West SK, Mabey D, Dawson CR, Bobo L, Bailey R, et al. Azithromycin in control of trachoma. Lancet. 1999;354(9179):630–5.
3. Keenan JD, Tadesse Z, Gebresillasie S, Shiferaw A, Zerihun M, Emerson PM, et al. Mass azithromycin distribution for hyperendemic trachoma following a cluster-randomized trial: A continuation study of randomly reassigned subclusters (TANA II). PLoS Med. 2018;15(8):e1002633.
4. Nash SD, Stewart AEP, Zerihun M, Sata E, Gessese D, Melak B, et al. Ocular chlamydia trachomatis infection under the surgery, antibiotics, facial cleanliness, and environmental improvement strategy in Amhara, Ethiopia, 2011-2015. Clin Infect Dis. 2018; 67(12):1840-6. https://doi.org/10.1093/cid/ciy377 PMID: 29741592
5. Burton MJ, Holland MJ, Makalo P, Aryee EAN, Alexander NDE, Sillah A, et al. Re-emergence of Chlamydia trachomatis infection after mass antibiotic treatment of a trachoma-endemic Gambian community: a longitudinal study. Lancet. 2005; 365(9467):1321-8. https://doi.org/10.1016/S0140-6736(05)61029-X PMID: 15823382

## Supporting information

S1 PRISMA Checklist 2009 PRISMA checklist. PRISMA, Preferred Reporting Items for Systematic reviews and Meta-Analyses.
(DOC)

S1 Protocol Discontinuation of mass azithromycin distribution for trachoma: a systematic review. Registered on PROSPERO (CRD42021140510).
(DOCX)

S1 PRISMA Flowchart 2009 PRISMA flowchart. PRISMA, Preferred Reporting Items for Systematic reviews and Meta-Analyses.
(DOC)
