# Supplementary material for: Stopping azithromycin mass drug administration for trachoma: A systematic review
Source: PLoS Negl Trop Dis. 2021 Jul 8;15(7):e0009491. doi: 10.1371/journal.pntd.0009491 (PMC8266061; doi:10.1371/journal.pntd.0009491)
Supplement: S1 Protocol — Registered on PROSPERO (CRD42021140510). (DOCX) [file pntd.0009491.s002.docx]

**TITLE**

Trachoma prevalence in the absence of mass azithromycin distribution: a systematic review

**MOTIVATION**

Trachoma, caused by repeated infections with ocular *Chlamydia trachomatis,* has an estimated disease burden of 3% of the world’s blindness and 84 million active cases [1]. Yet trachoma is a preventable and curable disease and may be the first bacterial disease to be eradicated in history. In many previously endemic regions of trachoma infection, prevalence has drastically declined in part due to mass azithromycin distribution in combination with water, sanitation, and hygiene interventions. The World Health Organization recommends continuing annual oral azithromycin treatment until affected regions drop below 5% prevalence of active trachoma (trachomatous inflammation –follicular, TF) in children aged 1-9 years. However, azithromycin only treats ocular chlamydia infection and the correlation between TF and infection has shown to be poor after repeated rounds of treatment [2-4]. Understanding what happens to active trachoma and ocular chlamydia prevalence in the absence of mass distributions of azithromycin (MDA) may guide future treatment and surveillance efforts. One 2008 study in northern Ethiopia found that discontinuation of MDA resulted in an increase in mean prevalence of trachoma infection in children from 8.3% at 0 months to 14.7% at 36 months [5]. This project describes a systematic review of existing evidence for community prevalence of both active trachoma and ocular chlamydia in the absence of azithromycin distribution.

In this systematic review and meta-analysis (should our data allow), we aim to review all studies reporting a pre-treatment time point and at least two post-treatment time points to note the prevalence of TF and chlamydia infection after discontinuing oral azithromycin treatment. We will include studies of any length, conducted at any time, in any country on community-wide distribution of oral azithromycin for the prevention and treatment of trachoma published in English through May 2020.

**REVIEW QUESTION(S)**

**1.** What is the prevalence of clinical trachoma (TF) and ocular chlamydia infection pre-MDA and post MDA?

**2.** Is there a correlation between pre-treatment trachoma prevalence and return of trachoma infection prevalence after discontinuing oral azithromycin treatment?

**SEARCH STRATEGY**

- **Search Items**
  - Trachoma
  - Azithromycin
- **Synonyms to be searched, if needed**
  - Azithromycin: Zithromax
- **Search algorithm**
  - ("Trachoma"[Mesh] OR trachoma) AND ("Azithromycin"[Mesh] OR azithromycin OR zithromax)
- **Databases searched and search details**
  - **MEDLINE**
    - Search bar
      - ("Trachoma"[Mesh] OR trachoma) AND ("Azithromycin"[Mesh] OR azithromycin OR zithromax)
- Full search details:
  - ("Trachoma"[Mesh] OR ("trachoma"[MeSH Terms] OR "trachoma"[All Fields])) AND ("Azithromycin"[Mesh] OR ("azithromycin"[MeSH Terms] OR "azithromycin"[All Fields]) OR ("azithromycin"[MeSH Terms] OR "azithromycin"[All Fields] OR "zithromax"[All Fields]))
- **EMBASE**
  - Search bar
    - ('trachoma'/exp OR trachoma) AND ('azithromycin'/exp OR azithromycin)
- **Web of Science**
  - Search bar (TOPIC)
    - ("Trachoma"[Mesh] OR trachoma) AND ("Azithromycin"[Mesh] OR azithromycin OR zithromax)
- **Cochrane Library**
  - Search bar (Title, Abstract, Keywords)
    - Trachoma AND azithromycin
- **Other sources**
  - **Abstracts**
    - Abstracts available online from the following conferences will be searched using the search terms outlined above:
      - American Society of Tropical Medicine and Hygiene (ASTMH)
      - Association for Research in Vision and Ophthalmology (ARVO)

**Article Eligibility Criteria**

- **Inclusion criteria**
  - Primary, quantitative data
  - Studies of community-wide oral azithromycin distribution for trachoma
  - Includes measurement of prevalence of ocular chlamydia and/or clinical trachoma before and after azithromycin distribution
  - Studies must include one pre-treatment time point and at least two post-treatment timepoints
  - Studies must be published in the English language
  - **Dates of inclusion:** All primary abstracts, reports, etc. published through 20 May 2020
- **Exclusion criteria**
  - Studies on mathematical modeling, surveillance reports, review articles
  - Studies without sufficient information on number of individuals/ villages tested
  - Studies without two distinct time points of pre- and post- azithromycin distribution
  - Studies on the use of azithromycin for purposes other than treatment of trachoma

*We will not exclude studies that concurrently use topical tetracycline for trachoma treatment

**OUTCOMES**

- **Primary outcomes of interest**
  - Prevalence of clinical trachoma pre-MDA and post-MDA
  - Prevalence of ocular chlamydia infection pre-MDA and post-MDA
- **Secondary outcomes**
  - Correlation between pre- and post-MDA prevalence of clinical trachoma
  - Correlation between pre- and post-MDA prevalence of ocular chlamydia infection

**DATA EXTRACTION**

- **Variables to extract**
  - Title
  - Author
  - Journal
  - Year of publication
  - Study design
  - Geographic location of study
  - Sample size (numbers of communities, numbers of individuals)
  - Pre-MDA trachoma prevalence (ocular chlamydia infection and/or clinical trachoma/TF)
  - Post-MDA trachoma prevalence (ocular chlamydia infection and/or clinical trachoma/TF)
  - Frequency of MDA
  - Duration of MDA

**Analyses**

- **Strategy for data synthesis (qualitative, quantitative)**
  - A qualitative synthesis of individual studies will be performed to discuss individual findings per the variables extracted.
  - For quantitative synthesis, a meta-analysis will be performed per the procedures outlined below
- **Meta-analysis (should our data have low heterogeneity)**
  - Estimates of prevalence pre- and post-MDA will be pooled across studies using a DerSimonian and Laird random effects model for overall prevalence of each outcome (clinical trachoma and ocular chlamydia infection)
    - Pre-MDA prevalence is predicting post-MDA prevalence
  - The I² statistic will be calculated as a measure of heterogeneity across studies. Sources of heterogeneity, including pre-MDA trachoma prevalence, country of origin, and year, will be evaluated using a random effects meta-regression model.
- **Quality Assessment**
  - Risk of bias will be assessed using the Cochrane risk of bias tool for randomized controlled trials and the ROBINS-I tool for non-randomized studies

**Registration Citation**

- PROSPERO Registration #: CRD42021140510

Available from:  <https://urldefense.proofpoint.com/v2/url?u=https-3A__www.crd.york.ac.uk_PROSPERO&d=DwIDAw&c=iORugZls2LlYyCAZRB3XLg&r=Ojl55QHvyscJEypoQg6cQoNr074Eo4Z7AYpbPyoIq1Q&m=tGe5ydZRf22CG4I-u8xjqyLn1ZBUMLk3IaEkA7ryZrI&s=5-myTuDFhJn6Ec7zn6pEFOaMSZJkikuFGVVCYutg8rY&e=> .

**References**

1. Who.int. [cited 2021 Feb 10]. Available from: <https://www.who.int/blindness/causes/trachoma/en/>
2. Lietman T, Porco T, Dawson C, Blower S. Global elimination of trachoma: how frequently should we administer mass chemotherapy? Nat Med. 1999;5(5):572–6.
3. Gill DA, Lakew T, Alemayehu W, Melese M, Zhou Z, House JI, et al. Complete elimination is a difficult goal for trachoma programs in severely affected communities. Clin Infect Dis. 2008;46(4):564–6.
4. Schachter J, West SK, Mabey D, Dawson CR, Bobo L, Bailey R, et al. Azithromycin in control of trachoma. Lancet. 1999;354(9179):630–5.
5. Keenan JD, Tadesse Z, Gebresillasie S, Shiferaw A, Zerihun M, Emerson PM, et al. Mass azithromycin distribution for hyperendemic trachoma following a cluster-randomized trial: A continuation study of randomly reassigned subclusters (TANA II). PLoS Med. 2018;15(8):e1002633.
