# Supplementary material for: Stopping azithromycin mass drug administration for trachoma: A systematic review
Source: PLoS Negl Trop Dis. 2021 Jul 8;15(7):e0009491. doi: 10.1371/journal.pntd.0009491 (PMC8266061; doi:10.1371/journal.pntd.0009491)
Supplement: S1 PRISMA Flowchart — PRISMA, Preferred Reporting Items for Systematic reviews and Meta-Analyses. (DOC) [file pntd.0009491.s003.doc]

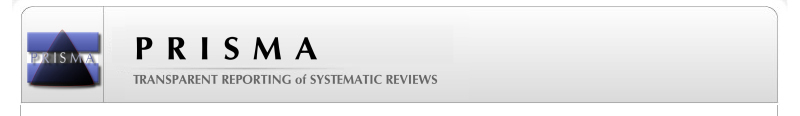
**2009 PRISMA Flow Diagram**

**Discontinuation of mass azithromycin distribution for trachoma: a systematic review**

**Screening**

**Included**

**Eligibility**

**Identification**

Records identified through database searching
(n = 1528)

Additional records identified through other sources
(n = 0)

Records after duplicates removed
(n = 884)

Records screened
(n = 884)

Records excluded
(n = 744)

Full-text articles assessed for eligibility
(n = 140)

Full-text articles excluded, with reasons
(n = 119)

Studies included in qualitative synthesis
(n =21)

Studies included in quantitative synthesis (meta-analysis)
(n = 0)
